# Iridoids and Other Monoterpenes in the Alzheimer’s Brain: Recent Development and Future Prospects

**DOI:** 10.3390/molecules23010117

**Published:** 2018-01-07

**Authors:** Solomon Habtemariam

**Affiliations:** Pharmacognosy Research Laboratories & Herbal Analysis Services, University of Greenwich, Central Avenue, Chatham-Maritime, Kent ME4 4TB, UK; s.habtemariam@herbalanalysis.co.uk; Tel.: +44-208-331-8302/8424

**Keywords:** monoterpenes, iridoids, Alzheimer’s disease, amyloid beta, drug likeness, multiple mechanisms

## Abstract

Iridoids are a class of monoterpenoid compounds constructed from 10-carbon skeleton of isoprene building units. These compounds in their aglycones and glycosylated forms exist in nature to contribute to mechanisms related to plant defenses and diverse plant-animal interactions. Recent studies have also shown that iridoids and other structurally related monoterpenes display a vast array of pharmacological effects that make them potential modulators of the Alzheimer’s disease (AD). This review critically evaluates the therapeutic potential of these natural products by assessing key in vitro and in vivo data published in the scientific literature. Mechanistic approach of scrutiny addressing their effects in the Alzheimer’s brain including the τ-protein phosphorylation signaling, amyloid beta (Aβ) formation, aggregation, toxicity and clearance along with various effects from antioxidant to antiinflammatory mechanisms are discussed. The drug likeness of these compounds and future prospects to consider in their development as potential leads are addressed.

## 1. Introduction

Alzheimer's disease (AD) is one of the most prevalent age-related diseases mostly affecting the elderly population. Of the estimated 5.5 million Americans with AD in 2017, 5.3 million comprising about 96% of the patients’ population, were 65 years of age or older [1]. The disease also accounts for up to 70% of all cases of dementia and has a global prevalence of about 47 million people in 2015 [2]. Moreover, the projected figure for dementia by the year 2050 is 131.5 million highlighting the rapid rate of increase in its importance. As life expectancy continues to increase all over the world in parallel with economic development, the risk of AD along with its cost and social burden will be felt even more in the future. The disease is characterized by progressive cognitive deficit and irreversible neuronal deterioration. To date, there is no cure for AD and the average lifespan between the manifestation of clinical symptoms and death is about 8.5 years [3]. The memory deficits in AD are also associated with behavioral changes making patient care management very challenging.

The therapeutic options for AD are very limited with drug therapy mainly directed at the cholinergic system by using cholinesterase inhibitors such as donepezil, galantamine, and rivastigmine. A limited benefit by using non-competitive *N*-methyl-d-aspartate (NMDA) receptor antagonists including memantine have also been employed [4,5]. In the recent review article, the role of natural products in ameliorating the various neurodegenerative diseases has been outlined [6]. Significant advances in understanding the therapeutic potential of polyphenolic compounds such as flavonoids [7,8,9,10,11,12], caffeic acid derivatives [13] and aromatic diterpenoids [14] that benefit AD through multiple mechanisms of actions have also been presented. One class of compounds that has not received much attention as potential therapy for AD is the monoterpene class. In the present communication, a systematic review of these compounds with special emphasis on iridoids is presented.

## 2. Overview of Iridoids Chemistry

**Figure 1:**
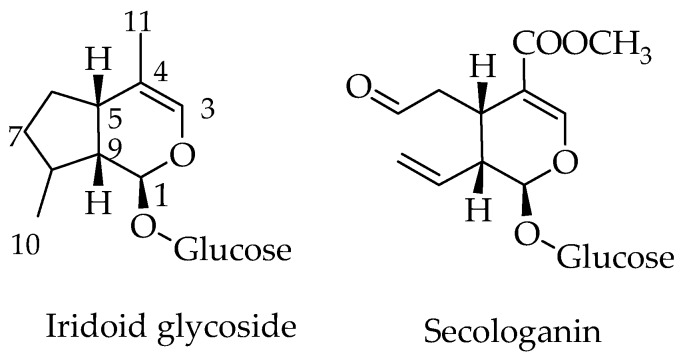
General structure of iridoids and secoiridoids.

Iridoids are the monoterpenoid class of natural products that are constructed with 10 carbon skeleton. One structural marker of this compounds is the *cis**-***fused *cy*clopenta[c]pyran system that exist in nature as glycosides, aglycones, in the form of secoiridoids or bisiridoids forms (Figure 1). In the case of secoiridoids, the C7–C8 bond of the iridoid skeleton is cleaved following a series of oxidation steps to give rise to compounds like secologanin (Figure 1), which also serves as a precursor to the synthesis of alkaloids. 

**Figure 2:**
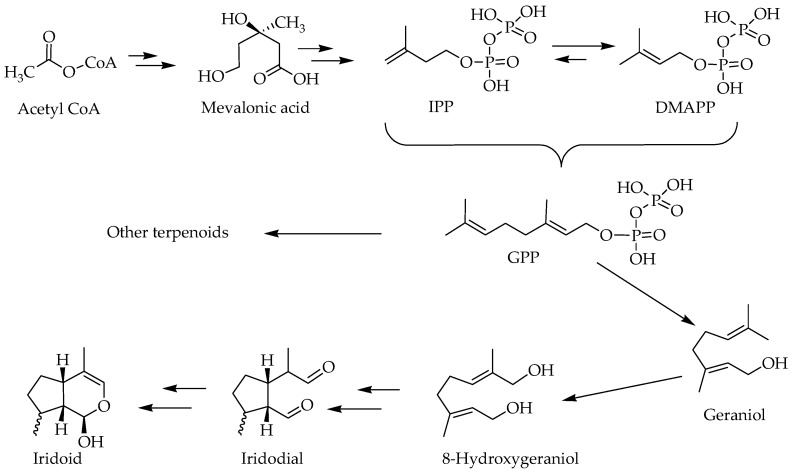
An overview of biosynthesis pathway of iridoids.

**Figure 3:**
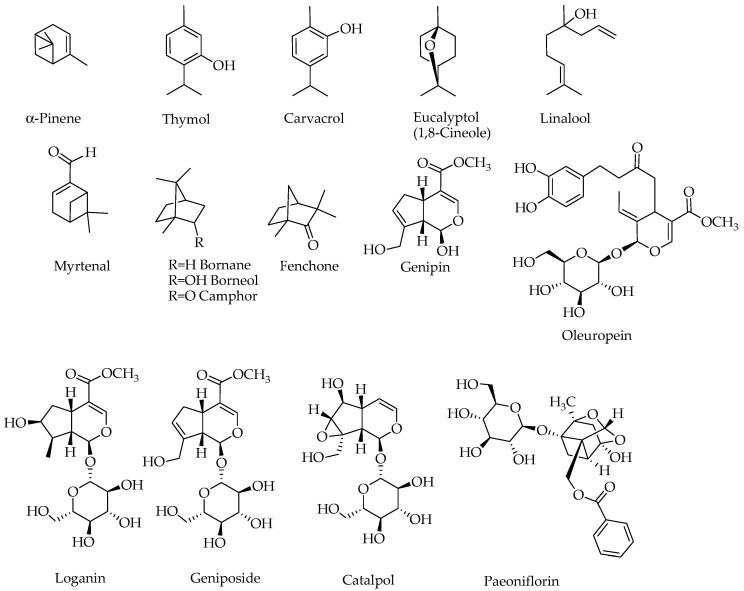
Structures of compounds with potential effects on the Alzheimer’s brain. Note that compounds containing a sugar moiety (oleuropein, loganin, geniposide, catalpol and paeoniflorin) are highly polar and hence are not components of essential oils.

The biosynthesis pathway of terpenoids has been reviewed in the various literatures [15,16] and involves some key biosynthetic intermediates like the mevalonic acid. Even though the starting primary metabolite goes as far back as a two-carbon metabolite, acetyl-CoA, the basic skeleton of all terpenoids is defined by the 5-carbon isoprene units in the form of isopentenyl pyrophosphate (IPP) and dimethylallyl pyrophosphate (DMAPP). The precursor of all terpenoids in the further steps of reaction is the geranyl pyrophosphate (GPP) that is made from two isoprene units (Figure 2). The sesquiterpenes (15 carbon), diterpenes (20 carbon) and triterpenes (30 carbons) are classical examples of terpenoids that arise from condensation of these isoprene units through a serious of enzyme-catalyzed reactions. The GPP gives rise to a range of cyclic and acyclic monoterpenes of biological significance [17], while the 8-hydroxygeraniol unique pathways leads to iridoids and their derivatives (Figure 2). The compounds scrutinized for their potential effect in ameliorating the biochemical and behavioral symptoms of AD are shown in Figure 3. 

## 3. General Function of Iridoids and other Monoterpenes in Nature

Why plants and animals produce secondary metabolites has been a century-old question that has not yet been fully answered. A number of general arguments presented in the last few decades have been based on the role of such compounds in cell-cell communication within the organism or plant-animal interactions including defense against pathogens [15,18,19,20]. With respect to chemical defense against herbivores and pathogens, the role of iridoids is well defined as these compounds have been demonstrated to be bitter and show good sets of biological activities [21,22]. Interestingly, animals such as butterfly are known to accumulate these chemicals as defense against pathogens [23,24,25]. The biological activities of iridoids in mammalian system have also been the subject of intense scrutiny in recent years and effects including antidiabetic properties have recently been reviewed along with other monoterpenes [17]. In this communication, the promise of monoterpenes, but primarily iridoids, (Figure 3) for treating AD is scrutinized by assessing published literature on their in vitro and in vivo effects. 

## 4. Therapeutic Potential for Alzheimer’s Disease

### 4.1. In Vitro Protective Effects

The vast arrays of neuroprotective effects of iridoids and some monoterpenes are shown in Table 1 [26,27,28,29,30,31,32,33,34,35,36,37,38,39,40,41,42,43,44,45,46,47,48,49,50,51,52,53,54,55,56]. The Aβ formation, aggregation and function have been the major target areas of AD for in vitro experiments. In a study by Marumoto et al. [26], the β-secretase (recombinant human BACE1) inhibitory activities of some monoterpenes have been evaluated. Even though the inhibitory activity of these compounds were confirmed, their activity was moderate (above 50 μM) with geranyl acetone being the most active (IC_50_ value of 51.9 ± 3.9 μM) followed by (+)-camphor (95.9 ± 11.0 μM), (−)-fenchone (106.3 ± 14.9 μM), (+)-fenchone (117.0 ± 18.6 μM), and (−)-camphor (134.1 ± 16.4 μM). A number of in vitro experiments have also been devoted to studying the inhibitory effects of monoterpenes against Aβ-induced cytotoxicity in neuronal cells in vitro. Treatment of cells with borneaol suppressed the Aβ-induced cytotoxicity and oxidative stress in the SH-SY5Y (human neuroblastoma) cells [31] while 1,8-cineole (eucalyptol) showed similar effect in PC12 (rat pheochromocytoma) cells [32]; and genipin in cultured hippocampal neurons [34]. The antioxidant activity of these monoterpenes is also evident from their ameliorating effect on the H_2_O_2_-induced oxidative stress as shown by catalpol in astrocytes [29], and α-pinene and 1,8-cineole in PC12 cells [33]. Cultured primary cortical neurons exposed to Aβ could also be rescued by geniposide from toxicity and oxidative stress [37]. In a further experiment to show the mechanism of action of geniposide in primary cultured cortical neurons’ protection, the geniposide-induced τ protein phosphorylation and phosphorylation of Akt at Ser-473 site and GSK-3β at Ser-9 site were shown to be inhibited by leptin antagonists [38]. The role of leptin as potential mechanism of iridoids action on the Alzheimer’s brain is discussed in detail in the following section. 

With respect to the Aβ-induced toxicity in the central neuronal cells, the role of insulin-degrading enzyme (IDE) has been highlighted in recent years. In addition to degradation and clearance of Aβ, the IDE play pivotal role in the regulation of Aβ activity. By using primary cortical neurons in a culture media, Zhang et al. [39] have demonstrated that geniposide enhance the phosphorylation of peroxisome proliferator-activated receptor γ (PPARγ). The effect of geniposide in the activation of the IDE promoter was also shown to be mediated via the glucagon-like peptide-1 (GLP-1) receptor while other pathways confirmed to be involved by inhibitor studies (see Table 1) where phosphatidyl inositol 3-kinase, PI3K, proto-oncogene tyrosine-protein kinase Src (c-Src), PPARγ, protein kinase A (PKA) and epidermal growth factor receptor (EGFR) [39,40]. Furthermore, in the SH-SY5Y cells, geniposide has been shown to ameliorate the cytotoxicity of Aβ along with its oligomer assembly and cytotoxicity [41]. The protective effect of geniposide in the SH-SY5Y cells treated with other toxicants such as formaldehyde has also been reported [42]. The effect of paeoniflorin in PC12 cells protection from Aβ was similar with geniposide in that its activity was correlated with upregulation of the protein kinase B (Akt) phosphorylation level, B-cell lymphoma 2 (Bcl-2) protein expression, reducing Bax protein expression and is inhibited by LY294002 [52]. The protective effect of paeoniflorin from the 6-hydroxydopamine-induced apoptosis in PC12 cells was also correlated with enhanced antioxidant capacity (GSH (glutathione - reduced form) level) and suppression of the nuclear factor kappa-light-chain-enhancer of activated B cells (NF-κB) translocation [53]. A number of other studies (Table 1) also showed the protective effect of paeoniflorin against Aβ cytotoxicity in PC12 cells [52,53,54] and SH-SY5Y cells [55]; as well as glutamate-induced cytotoxicity in PC12 cells [56].

Hydrogen peroxide (H_2_O_2_)-induced cytotoxicity in PC12 cells could be inhibited by geniposide through the PI3K-dependent pathway as evidenced from the study using a selective inhibitor, LY294002 [43]. In the same cell system, Liu et al. [44] also showed that the effect of geniposide in reversing the oxidative stress induced by H_2_O_2_ involves an increased level of Bcl-2 by activation of the mitogen-activated protein kinase (MAPK), mitogen-activated protein kinase kinase (MEK) and rapidly accelerated fibrosarcoma proto-oncogene serine/threonine-protein (c-Raf) phosphorylation along with the phosphorylation of the p90 variant of the ribosomal s6 kinase (p90RSK) [43]. The requirement of the PI3K and GLP-1 receptor activation has also been confirmed in the PC12 cells protection from the H_2_O_2_-induced cytotoxicity [44].

The antiinflammatory effect of these compounds in the CNS came from evidences in vitro showing the inhibition of nitric oxide (NO) release from the lipopolysaccharide (LPS)-stimulated microglia by genipin along with suppression of microglial cells activation [34]. Beyond suppression of NO production, genipin also ameliorated the LPS-induced tumour necrosis factor-α (TNF), interleukin-1β (IL-1), prostaglandin E2 (PGE-2), intracellular reactive oxygen species (iROS), and NF-kB activation in microglial cells in vitro [35].

In an organotypic cultured hippocampal tissues, the scopolamine-induced functional changes was shown to be inhibited by loganin along with inhibition of acetylcholinesterase (AChE), butyrylcholinesterase (BChE) and β-secretase (BACE1) [45]. An effect on β-secretase (BACE1) inhibitory activity of loganin has also been reported by Youn et al. [49]. A direct effect on one of the most prevalent AD target, AChE, for loganin with IC_50_ value in sub-micromolar range was particularly impressive [46]. A further molecular docking studies have shown that loganin’s non-competitive type of interaction generate a negative binding energies for cholinesterase as well as BACE1 suggesting a high affinity and tighter binding capacity for the active site of the enzymes [46]. As BChE (though to a lesser extent, see Table 1) is also inhibited, loganin appear to target AChE, BChE, and BACE1 that are all important in AD pathology. The Aβ-induced inflammatory changed in PC12 cells could also be inhibited by loganin as evidenced from a reduction in the level of TNF-α and protein expression of iNOS and cyclooxygenase-2 (COX-2) [47,48]. These effects were also correlated with inhibition of NF-κB along with the closely related regulatory pathways including the phosphorylation of MAPKs (ERK1/2 (Extracellular signal–regulated kinase ½), p38 and JNK (c-Jun N-terminal kinase) [47]. 

A number of other studies (Table 1) have shown that monoterpenes possess direct inhibitory effect against AChE activity. This includes a report by Kaufmann et al. [50] on 8-cineole, carvacrol, myrtenal and verbenone, although the best activity in this study was observed at relatively high concentration (IC_50_ = 170 μM for myrtenal). On the other hand, oleuropein, thymol and carvacrol have been shown to have a much better activity but the best activity (IC_50_ < 5 μM) was obtained when a carbamate moiety was added to carvacrol through a synthesis approach [51]. In the latter case, there has also been a drive to improve the biological activity of existing anti-Alzheimer’s drugs by incorporating the monoterpene skeleton through synthesis. For example, with the help of a docking-based design, galantamine-camphane hybrids have been shown to display over a 100-fold better activity in AChE inhibition than galantamine [27].

All the in vitro data is shown in Table 1, which clearly indicates the therapeutic potential of iridoids as well as other monoterpenes in AD. The gross inhibition of cytotoxicity in neuronal cells induced by Aβ and other toxic agents have been demonstrated to be ameliorated. The reactive oxygen species (ROS), proinflammatory cytokines and many mediators could also be suppressed while mitochondrial deterioration was inhibited. At the molecular level, a range of antioxidant proteins and enzymes could be enhanced by these natural products along with anti-apoptotic genes and proteins, while proapoptotic genes and proteins appear to be suppressed (Table 1).

### 4.2. Evidence of Efficacy Demonstrated through In Vivo Studies

In parallel with the overwhelming in vitro data, animal studies on iridoids and some other monoterpenes (Table 2) have shown potential therapeutic effects for treating AD [57,58,59,60,61,62,63,64,65,66,67,68,69,70,71,72,73,74,75,76,77]. The neuroprotective effect of carvacrol in vivo was studied by Zhong et al. [55] using the intracerebral hemorrhage mouse model, where a significant reduction of the aquaporin-4 (AQP4)-dependent oedema was observed. It is worth noting that AQP4 is a water channel in the brain that plays major role in the development of cerebral oedema. The structural, physiological and pathological significance of ACQ4 has been extensively reviewed [78,79,80,81,82]. Considering the pathophysiological role of AQP4 in a range of CNS disorders including ischemic stroke [83], neuroinflammation [84] and autoimmune neurodegenerative diseases [85], the reversal of cerebral oedema induced through AQP4 activity by monoterpenes is an interesting observation.

A number of studies have targeted the oxidative stress and associated disorders by inducing the pathology with d-(+)-galactose injection into experimental animals. In this model, catalpol has been shown to reduce the level of Aβ in the cerebral cortex along with improvement of learning and memory; while the level of antioxidant defenses (SOD and GPx) were boosted [58]. In senescent mice treated with _D_-galactose, Zhang et al. [60] also reported neuroprotection by catalpol as evidenced by the increased level and activity of choline acetyltransferase (CHAT). Moreover, catalpol in this model has been shown to reverse the suppressed level of muscarinic acetylcholine (ACh) receptor M1 while concomitantly suppressing the level of inflammatory and oxidant markers (TNF-α, IL-1 and advanced glycation end products (AGEs)) [60]. Improvement of memory deficit along with antioxidant markers (glutathione S-transferase (GSH-ST), glutamine synthetase (GS) and creatine kinase (CK) have also been shown for catalpol [61,63].

In other experiments, Aβ was directly injected into the brain to study the biochemical and behavioral changes in animals. Catalpol was among the iridoids showing activity in this model where prevention of the ACh neuronal damage was noted from the increased level of choline CHAT positive cells density in cerebral cortex as well as increased level of ChAT activity [28]. Geniposide also ameliorated the Aβ-induced neuronal abnormalities including cellular densities and synaptic proteins level in the transgenic mice model [63]. On the other hand, linalool has been shown to reverse cognitive deficits and altered the level of the antioxidant and protein (SOD, GPx, AChE) levels/activity in mice injected with Aβ [69]. The effect of paeoniflorin in memory improvement and protection of animals from Aβ through mechanisms including enhancing antioxidant defenses (e.g., GSH) and calcium homeostasis have also been reported [76,77]. 

Zhang et al. [62] employed the APP/PS1 Transgenic mouse model of AD to study the potential benefit of geniposide. The insulin deficiency induced by streptozotocin (STZ) in these wild-type transgenic animals appeared to enhance the GSK-3β level/activity which was suppressed by geniposide administration in a dose dependent manor. It is worth noting that the doses employed here were very small (5, 10, and 20 mg/kg). The data were also in line with the broader effect of geniposide in signal transduction pathways related to insulin resistance reviewed recently [17]. The GSK-3β plays direct role in τ protein hyperphosphorylation [86,87]. The role of the Akt in the regulation of GSK-3β is also well understood and its phosphorylation initiates its inactivation that appeared to be modulated by geniposide. In agreement with this data, geniposide can also regulate the phosphorylation of τ protein both in the insulin-dependent and independent manor in primary cultured cortical neurons [63]. It does also enhance the phosphorylation of Akt at Ser473 and Thr308 sites [63]. The dual effect of geniposide both in diabetes and AD is thus evident from its effect on the phosphorylation of τ protein via the PI3K-GSK-3β kinase pathway. To date, hyperphosphorylated τ protein is one of the pathological hallmark of AD as it is the principal component of neurofibrillary tangles (NFTs) [87]. The structural integrity of τ protein is regulated by a cascade of phosphorylation-related pathways, and hence both kinases and phosphatases play important roles in stable NFT formation. The GSK-3β being the key player in the kinase—mediated hyperphosphorylation of τ protein, its regulation by geniposide seems to shed some light into the possible mechanism of iridoids’ action. The crosstalk between diabetes and AD was also highlighted by Gao et al. [67] who confirmed the potential role of geniposide through GSK-3β regulation. Similarly, in the study by Liu et al. [68], geniposide has been shown to decrease the Aβ1-42 level while improving the expression of IDE in Aβ-treated STZ-induced diabetic rats. In the further experiment on transgenic mice model, geniposide was shown to improve learning and memory along with antiinflammatory effect (through suppression of RAGE-dependent signaling in activation of ERK and IκB/NF-κB and the production of TNF-α, IL-1β) and lowering the Aβ level in the cerebrum [65]. Other compounds which have been shown to improve learning and memory in transgenic model of AD include linalool that could suppress pro-inflammatory proteins such as p38 MAPK, NOS-2, cyclooxygenase-2 (COX2) and IL-1β [70]. The effect of paeoniflorin in the transgenic mouse model of AD was also studied by Gu et al. [75]. In addition to improvement of the memory deficit, a reduction in the level of inflammation (NF-κB, TNF-α, IL-1β, IL-6) and apoptotic (caspase-3) markers were observed. As demonstrated for geniposide (above), paeoniflorin also modulate the GSK-3β signaling in transgenic animal model of AD [75].

Other behavioral models of AD included the scopolamine-induced AD model where loganin showed beneficial effect through the route of administration [71]. The neuroprotective effect of monoterpenes in other in vivo models has also been documented. For example, oleuropein could ameliorate the pentylenetetrazole (PTZ)-induced seizures in mice or colchicine-induced learning and memory deficits [73]. 

## 5. Insights into the Mechanism of Action of Iridoids and Other Monoterpenes in AD

**Figure 4:**
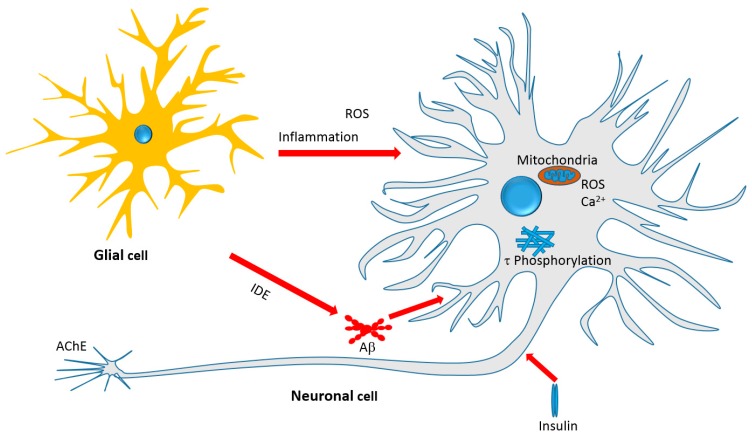
Therapeutic targets of iridoids and other monoterpenes discussed in this review. Antiinflammatory effect, amelioration of oxidative stress, mechanisms related to Aβ formation, aggregation and clearance, τ-protein phosphorylation and aggregation, and neurotoxicity associated with mitochondrial dependent and independent mechanisms are among the therapeutic targets.

The previous sections on the in vitro and in vivo effects of monoterpenes provided a plethora of evidences linking these compounds with key pathological pathways of AD. The general mechanism of action of monoterpenes in the AD brain is depicted in Figure 4. Some of the key features of monoterpenes, particularly iridoids, as an emerging class of compounds as anti-AD agents are shown below.

The role of Aβ in the pathology and as therapeutic target for AD has been reviewed in the various literatures (e.g., [88,89,90]). Recent review articles from our laboratories have also shown that many polyphenolic compounds such the flavonoids, diterpenoids and cinamate derivatives display therapeutic potential for AD through multiple mechanisms involving Aβ [6,7,8,9,10,11,12,13,14]. Hence, the formation, aggregation and toxicity of Aβ can all serve as targets for therapeutic agents. The direct role of monoterpenes in the formation and aggregation of Aβ is however less clear and the observed activity at moderate concentration may not be of a high degree of therapeutic relevance. Never the less, direct effect on APP processing enzymes has been shown. The predominant forms of the pathological Aβ in the brain are Aβ1–40 and to a lesser extent Aβ1–42 which are formed through the amyloidgenic β-secretase-dependent pathway. The selective inhibition of this enzyme by monoterpenes (Table 1) without much effect on the non-amyloidogenic marker enzyme (α-secretase) is an interesting finding. A large body of evidence also suggests that monoterpenes (Table 1 and Table 2) ameliorate the Aβ-induced cytotoxicity both in cultured neuronal cells and various animal models of AD [6,7,8,9,10,11,12,13,14]. Upon aggregation, the Aβ oligomers induce neurotoxicity leading to cell death, impairment of synaptic function and behavioral deficits that are commonly observed in AD animal models. Hence, one major target of the iridoids as well as the selected other monoterpenes appear to be mediated through mechanisms related to Aβ formation and/or toxicity.

The role of ROS in Aβ-induced neurotoxicity has been well established from evidences mostly linking redox metals like copper, zinc, and iron coordinating the generation of toxic free radicals and/or ROS [91,92,93,94,95,96,97]. As inhibitors of ROS generation through direct metal chelation and ROS scavenging, the role of polyphenols as potential therapeutic agents for AD has been extensively studied. In this direction, our own studies on catechol functional group and the flavonoid skeleton as optimized structural moieties for biological effects have been exhaustively researched [98,99,100,101,102,103,104,105,106,107,108,109,110,111,112,113,114,115]. The monoterpenes presented in this communication however lack such structural moiety unless additional skeleton as that shown in oleuropein is added (Figure 3). Their effect on the amelioration of the Aβ-toxicity as well as neurotoxicity induced by H_2_O_2_ suggest a mechanism of action beyond direct ROS scavenging. This can include boosting antioxidant defenses, and in this connection, numerous studies have shown an increased antioxidant status in the AD brain following treatment by monoterpenes (Table 2). 

Another well-defined mechanism of action of monoterpenes in the AD brain appears to be linked to anti-inflammatory effect. In view of neuroinflammation as the major pathological hallmark of AD, the role of inflammatory cells activation in the brain, primarily astrocyte and microglial cells, have been investigated in the last few decades. Readers are thus directed to excellent reviews in the field [116,117,118,119,120,121,122]. Interestingly, all of the best-characterised inflammatory markers such as TNF, IL-1, COX and NOS have been shown to be suppressed by the studied compounds in this review. Since inhibition of these proinflammatory cytokines such TNF is known to provide favorable outcome in AD [123,124], the suppressive effect of numerous monoterpenes on proinflammatory level in the Alzheimer’s brain is in line with potential benefit in AD. Among the regulators of cytokines in their proinflammatory effect is the NF-κB which has been demonstrated to play key role in AD [125]. As modulators of the NF-κB, monoterpenes appear to also link their potential therapeutic mechanism through such an effect. 

Leptin is one of the hormones produced by adipocytes with primary function in body weight and fat regulation through diverse mechanisms including modulation of food intake and metabolism [126]. Diverse other functions of leptin were however emerging in recent years; these include modulation of the immune response and broad range of neuronal regulation from neuroprotection to cognition [127,128]. The role of leptin receptor-mediated regulation in the cerebral cortex and hippocampus and dysregulation in AD has also been well recognized [129,130,131,132]. In addition to neurons, immune cells in the brain such as astrocytes and glial cells do also express leptin receptors and are regulated by this adipocytes’ hormone [133,134]. Considering evidences showing the potential neuroprotective effect of leptin under pathological condition as well as many other in vitro and in vivo experiments (e.g., [135,136,137]), the modulatory effects of monoterpenes in this system is an exciting development. As leptin antagonist abolished the effect of geniposide on τ phosphorylation and phosphorylation of Akt at Ser-473 site and GSK-3β at Ser-9 in the Alzheimer’s brain (Table 1), part of the iridoids action is likely to be mediated through leptin regulation. 

As with their formation, the degradation of Aβ peptides and plaques must be tightly regulated to avoid pathological disorders such the AD. Among the various mechanisms involved in Aβ degradation and clearance include the Aβ proteases, low-density lipoprotein receptor-related protein 1, and the apolipoprotein E systems [138]. Of the protease enzymes, neprilysin (also known as membrane metallo-endopeptidase) is a zinc-dependent metalloprotease that cleaves Aβ and have shown a good correlation with Aβ accumulation [139]. The endothelin-converting enzyme, and angiotensin-converting enzyme do also function as Aβ degrading enzymes. The role of IDE in Aβ degradation has recently been clarified and its dysregulation is now known to contribute to the pathology of the AD [138,140,141]. In fact, IDE is considered to be the main extracellular protease enzyme for the degradation of Aβ [142,143] and its expression, as with neprilysin, in the hippocampus has been shown to decrease with increasing age [144]. Hence, upregulation of the Aβ degrading enzymes is among the therapeutic approaches for AD [145,146,147]. In the brain, glial cells such as the microglia and astrocytes are the main source of IDE secretion [113] and their dysregulation could thus contribute to AD pathology; while promotion of IDE secretion from these cells could be implicated in AD therapy through enhancing Aβ clearance. The astrocytes and microglial cells are also primary phagocytes in the brain that recognize Aβ through membrane receptors to remove through phagocytosis [148]. The therapeutic approach of AD by upregulating IDE is however a tricky one, as IDE also selectively degrades insulin and its inhibitors are needed to improve glucose homeostasis (e.g., in diabetes). The role of iridoids in this regards is very interesting as geniposide has been shown to upregulate IDE [39] while displaying potent antidiabetic effect [17]. As IDE is degrading the monomeric form of Aβ, it is preventing the formation of oligomers or aggregates that is prerequisite to Aβ cytotoxicity in neuronal cells. Hence, a clear line of evidence is now available for geniposide and/or other iridoids that showed a promise in the Alzheimer’s brain. 

The dual effect of iridoids in diabetes and AD is also manifested from the possible mechanism of action related to the τ protein phosphorylation pathway. The formation of intracellular NFTs is a result of aggregation of the hyperphosphorylated τ-protein. As a major component of the neuronal cytoskeleton, τ-protein is closely associated with microtubules and aids a number of neuronal functions from axonal transport to neurite outgrowth [87,149]. The function of τ protein in stabilizing the microtubule to facilitate the normal neuronal function is governed by its phosphorylation which is regulated by a number of cellular kinases and phosphatases [150]. Consequently, τ protein dysregulation is among the pathological hallmark of AD as in NFTs and hence serves as a target for drug therapy. Hyperphosphorylation of τ-protein quickly initiates the formation of helical filaments and aggregates as seen in the NFTs of AD. This intern leads to microtubule disassembly and destabilization [151]. The signaling cascade in τ-protein hyperphosphorylation has been shown to involve the GSK-3β that directly act on the protein (to phosphorylate it) and make it to disassociate with the microtubules [152,153]. Hence, downregulating GSK-3β by drugs is essential in AD not only to regulate τ-protein hyperphosphorylation but also to manage other deleterious effect of GSK-3β such as in ROS generation from the mitochondria. For example, GSK-3β has been shown to down-regulate the transcription factor Nrf2 after oxidative damage [154]. The GSK-3β itself is regulated by other kinases such as the Akt that phosphorylate GSK-3β at different sites to negatively regulate its activity. Furthermore, activation of PI3K triggers the activation of Akt that phosphorylates GSK-3β leading to inhibition of τ-protein phosphorylation. Hence, the dysfunction of PI3K/Akt signaling is linked to τ-protein phosphorylation or NFT formation in AD. The p38 MAPK is also emerged as anther kinase involved in τ-protein phosphorylation and hence can be targeted by drugs [155,156,157]. A review article of such signal transduction pathways and possible pharmacological regulations is eloquently presented by Medina et al. [158]. The observation of iridoids to regulate τ-protein phosphorylation by inhibiting GSK-3β and regulation of the associated system primarily the PI3K/Akt signaling (Table 1 and Table 2) is a remarkable documentation of record for this group of compounds. The pioneering compound in this regard is geniposide (e.g., [38,39,67]). Other natural products such phenolics including resveratrol [159], curcumin [160], hyperforin [161] and capsaicin [162] have been shown to display inhibitory effect against τ protein hyperphosphorylation as well as affect in vivo models of AD. Hence, iridoids with structural feature distinctively different from polyphenols appear to share one common feature of mechanism in their potential AD modulations. 

Overall, it appears that the iridoids and some other monoterpenoids target the various cellular and biochemical features of AD pathology depicted in Figure 4. They target oxidative stress by boosting antioxidant defenses; inhibit the Aβ cascades particularly neurotoxicity; inhibit τ-protein phosphorylation and hence NFTs formation; promote the clearance of toxic proteins (Aβ) through IDE; modulate the insulin signaling pathway and insulin resistance as antidiabetic agents; and display a range of anti-inflammatory effects by suppressing the expression of numerous key proinflammatory proteins. Another interesting development is the direct effect of monoterpenes on AChE enzyme and further possible opportunity of potency optimization through chemical synthesis.

## 6. Drug-Likeness and Structural Perspectives

A range of qualitative and quantitative measures of drug-likeness parameters have been employed in recent years to identify leads in drug discovery researches as well as improving the efficiency of known bioactive compounds. In the in silico drug-likeness predictions, the undesirable properties of small molecular weight compounds assessed by poor ADMET (absorption, distribution, metabolism, excretion, and toxicity) characteristics are used as a screening tool [163]. In this regard, monoterpenes (unless glycosylated, see Figure 3) act as a component of essential oils with list solubility profile in water falls within the poor drug-likeness profile. Accordingly, their absorption, distribution, metabolism, and excretion profiles were not in line with what one expects as ideal drug molecules. Hence, all in vitro and in vivo data so far suggest that they are absorbed and distributed to tissues but with far slower rate than that ideally expected [164,165,166,167,168]. Human trial also confirmed these observations but the iridoid glycosides, with sugar attachment thereby increasing their polarity, appear to be a good compromise in vivo [169,170,171,172,173]. Like many other sugar-linked natural products, the iridoid glycosides such as geniposide have been shown to be metabolized by intestinal bacteria to release their aglycone (e.g., genipin) [174,175] which also give rise to conjugated products (e.g., with glucuronic acid) [176]. Increasing water solubility by glycosylation to release a bioactive aglycone in the intestine has been reported to be one way of enhancing bioavailability for natural products [177]. Even for glycosides such as geniposide, however, the absolute oral bioavailability after oral administration remains to be poor ~9.67% [178]. Nevertheless, both in vitro and in vivo experiments have shown good effects in ameliorating the biochemical and behavioral markers of AD. Hence, despite their predicted poor drug-likeness profile, iridoids and other monoterpenes have shown potent activity to be seriously considered as potential lead compounds in future studies. 

## 7. Future Prospects

One common advantage of employing compounds of natural origin (e.g., monoterpenes) is that they are associated with common foods and beverages that are already in use for human consumption. As neuromodulators, particularly in AD, the beneficial effects of some essential oils as crude mixtures of small molecular weight fragrant compounds including monoterpenes have been reported in the various literature [see review article, 179]. As indicated in the preceding section, however, the drug likeness of these molecules has not been in favor of their development as drugs given their poor water solubility and bioavailability. The iridoids glycosides appear to offer a better bioavailability profile and pharmacology as evidenced from their activity profile in vitro and in vivo. The fact that both the glycosylated and the aglycones are active in vitro suggests that the glycosides being a better bioavailable compounds could be more preferable as drug candidates. One should bear in mind that research on this class of compounds is still at its infant stage and more work is needed on optimization of their pharmacology through medicinal chemistry. The effect of some monoterpenes, for example, could be enhanced by over 100-fold when other functional groups such as a carbamate moiety were added or they being incorporated into the existing anti-AD drugs such as galantamine [27,51]. Naturally, human clinical trials would offer not only valuable data on efficacy but also pharmacokinetic profile that are desperately needed for these compounds. Such study of course would be preferred once a lead compound is identified and optimized through future research. In the meantime, all the available data now suggest that small molecules of the iridoids class and related monoterpenes could be considered as potential leads for AD therapy. 

## Figures and Tables

**Table 1:** In vitro effects of iridoids and other monoterpenes related to AD pathology.

Compound	Model	Outcome	Reference
Geranyl acetone; (+)-camphor; (−)-fenchone; (+)-fenchone; (−)-camphor	β-secretase (recombinant human BACE1)	Moderate activity with inhibitory concentration (IC_50_) higher than 50 μM.	[26]
Bornane (or camphane)—hybrids of Galantamine	Docking-based design and synthesis of galantamine-camphane hybrids.	Hybrids showed over 191—better inhibition of AChE than galantamine	[27]
Catalpol	PC12 cells—Aβ25-35	10 and 100 μL—Increase expression and activity of ChAT.	[28]
Catalpol	H_2_O_2_-induced oxidative stress in astrocytes; primary cultures in mice.	50–500 μM—Increase cell viability; reduce the intracellular level of ROS; suppress oxidative stress by restoring the activities of antioxidant enzymes (GPx, GR and GSH); no effect on CAT activity.	[29]
Catalpol	CHO cells	10 and 100 μM—No effect on AChE activity; elevate the M-2 receptor density but did not occupy the M receptor binding site.	[30]
(−)- and (+)-Borneol	Aβ—induced oxidative stress in SH-SY5Y cells	100 μM—Inhibit cytotoxicity; decrease ROS generation; increase HO-1 and nuclear translocation of Nrf2 expressions; increase Bcl-2 while decreasing Bax expression.	[31]
1,8-cineole (eucalyptol)	Aβ(25-35) treated PC12 cells	Restored cell viability; reduce mitochondrial membrane potential, ROS and NO levels; suppress the levels of proinflammatory cytokines (TNF-α, IL-1β and IL-6); lower the expression of NOS-2, COX-2 and NF-κB.	[32]
1,8-Cineole and α-Pinene	H_2_O_2_-induced oxidative stress in PC12	Inhibit the level of iROS; enhance the expression of antioxidant enzymes (CAT, SOD, GPx, GR and HO-1. Decrease apoptosis (reduce capase-3 activity); induce the nuclear Nrf2 factor.	[33]
Genipin	Cultured hippocampal neurons treated with Aβ-25-35	20–40 μM—Reduce LDH release; improve morphological appearance.	[34]
Genipin	Cultured rat brain microglial cells treated with LPS	5–20 μM - Inhibit NO release; suppress the level of TNF, interleukin-1β, PGE-2, iROS; suppress NF-κB activation; reduce NO release stimulated by IF-γ and Aβ.	[35]
Genipin	A23187 (a calcium ionophore)-induced cytotoxicity in neuro2a cells	8 and 20 μM—Cytoprotective effect from caspase 3/7 and ER stress.	[36]
Geniposide	Cultured primary cortical neurons treated with Aβ	Reverse mitochondrial dysfunction by recovering ATP generation, MMP, and cytochrome c oxidase and caspase 3/9 activity; reduce ROS production and cytochrome c leakage; inhibit apoptosis.	[37]
Geniposide	Rat primary cultured cortical neurons	Decrease the phosphorylation of τ protein while inducing the phosphorylation of Akt at Ser-473 site and GSK-3β at Ser-9 site; effect could be prevented by leptin antagonist.	[38]
Geniposide	Primary cortical neurons and PC12 cells—insulin-degrading enzyme (IDE) in the degradation and activity of (Aβ)	Up-regulation of IDE by geniposide inhibited by LY294002 (inhibitor of PI3K), PP1 (inhibitor for c-Src), GW9662 (antagonist of PPARγ), H89 (inhibitor of PKA) and AG1478 (antagonist of EGFR). Enhanced the phosphorylation of PPARγ; accelerate the release of phosphorylated FoxO1 (forkhead box O1) from nuclear fraction to the cytosol; directly activate the activity of IDE promoter in PC12 cells.	[39,40]
Geniposide	SH-SY5Y cells treated with Aβ	5–200 μM—Decrease cytotoxicity by remodeling Aβ assembly.	[41]
Geniposide	SH-SY5Y treated with formaldehyde	Restore normal morphology and inhibit apoptosis in dose dependent manor; increase the activity of intracellular antioxidants (SOD and GPx); increase antiapoptotic gene Bcl-2 while downregulating the expression of apoptotic-related gene (P53, caspase 3 and caspase 9).	[42]
Geniposide	H_2_O_2_-induced cytotoxicity in PC12 cells	Induce the expression of the antiapoptotic protein Bcl-2; inhibit apoptosis; effect dependent on PI3K (inhibited by LY294002); enhance the phosphorylation of Akt308, Akt473, GSK-3β and PDK1 under oxidative stress.	[43]
Geniposide	H_2_O_2_-induced cytotoxicity in PC12 cells	25–100 μg/mL—Increase the expression of anti-apoptotic proteins (including Bcl-2 and HO-1); effect inhibited by LY294002; increase Bcl-2 level by activation of MAPK, MEK and c-Raf phosphorylation; effect inhibited by U0126 (inhibitor of MEK).	[44]
Loganin	Organotypic cultured hippocampal tissues	Increased the total activity of fEPSP after high frequency stimulation.	[45]
Loganin	AChE, BChE, and β-site amyloid precursor protein cleaving enzyme 1 (BACE1)	AChE inhibitory effects with IC_50_ values for AChE and BChE of 0.33 and 37.78 μM, respectively.	[46]
Loganin	Aβ25-35-induced inflammatory damage in PC12 cells	Inhibit cytotoxicity by suppressing ROS generation; inhibit apoptosis by suppressing caspase-3 activity and regulating cell cycle; suppress the level of TNF-α and protein expression of iNOS and COX-2; inhibit NF-κB activation by modulating degradation of the inhibitory subunit IκB; inhibit phosphorylation of MAPKs (ERK1/2, p38 and JNK).	[47,48]
Loganin	β-Secretase (BACE1)	92 μM—Inhibit BACE1 with little effect on α-secretase.	[49]
Myrtenal	Anti-acetylcholinesterase activity	1,8-cineole, carvacrol, myrtenal and verbenone AChE; the highest inhibitory activity was observed for myrtenal (IC_50_ = 170 μM).	[50]
Thymol and carvacrol derivatives with added carbamate moiety—Synthesis	Acetylcholinesterase and butyrylcholinesterase inhibition assay	5-isopropyl-2-methylphenyl(3-fluorophenyl)carbamate was found to be the most potent AChE inhibitor with IC_50_ values of 2.22 μM; 5-isopropyl-2-methylphenyl (4-fluorophenyl)carbamate exhibited the strongest inhibition against BuChE with IC_50_ value of 0.02 μM.	[51]
Paeoniflorin	Aβ25-35-induced PC12 cell injury	10 μM—Inhibit cytotoxicity; upregulate AKT phosphorylation; increase Bcl-2 protein expression, reduce Bax protein expression and caspase-3 activation. Effect reversed by LY294002.	[52]
Paeoniflorin	6-Hydroxydopamine-induced apoptosis in PC12 cells	30–300 μM—Suppresses mitochondria-mediated apoptosis; increase GSH level; attenuate the 6-OHDA-induced NF-κB translocation without affecting phosphorylation of Akt, JNK, p38, and ERK1/2; blocked the induced protein kinase Cδ (PKCδ) upregulation.	[53]
Paeoniflorin	Aβ25-35-induced neurotoxicity in PC12 cells	2–50 μM—Attenuate cytotoxicity mediated through mitochondrial dysfunction (decreased mitochondrial membrane potential, increased cytochrome c release as well as activity of caspase-3 and caspase-9).	[54]
Paeoniflorin	Aβ25-35-induced cytotoxicity in SH-SY5Y cells	Restore cell viability; inhibit apoptotic and ROS production; inhibit mitochondrial dysfunction (mitochondrial membrane potential, increased Bax/Bcl-2 ratio, cytochrome c release and activity of caspase-3 and caspase-9).	[55]
Paeoniflorin	Glutamate-induced cytotoxicity in PC12 cells	0.1–10 μM—Protect cells from cytotoxicity; up-regulate Bcl-2 and down-regulate Bax.	[56]

Bax, Bcl-2-associated X protein (bcl-2-like protein 4); CAT, Catalase; EGFR, epidermal growth factor receptor; ER, endoplasmic reticulum GPx, glutathione peroxidase; GR, glutathione reductase; GSH, glutathione - reduced form; GSK-3β, glycogen synthase kinase 3β; IF-γ, interferon-γ; iNOS, inducible nitric oxide synthase; iROS, intracellular ROS; LDH, lactate dehydrogenase; MMP, mitochondrial membrane potential; NOS, nitric oxide synthase; Nrf2, nuclear factor erythroid 2; PDK1, 3-phosphoinositide-dependent protein kinase-1; fEPSP, field excitatory postsynaptic potential.

**Table 2:** In vivo effects of iridoids and some other monoterpenes as potential modulators of AD.

Compound	Model	Outcome	Reference
Carvacrol	Bacterial collagenase-induced intracerebral hemorrhage mouse model - Single doses of 10, 25, 50 or 100 mg/kg, i.p.	Improve neurological deficits; reduce cerebral edema and Evans blue leakage; decrease AQP4 mRNA in a dose-dependent manner; reduce AQP4 protein expression in the perihematomal area.	[57]
Catalpol	_D_-(+)-galactose mice model—20 mg/kg, intragastric for 30 days	Reduce the oxidative stress in the cerebral cortex; regulate the activities and concentration of SOD, glutathione peroxidase and catalase (MDA level not altered); reduce the levels of soluble Aβ40 and Aβ42 in the cerebral cortex; effects regulated by IDE; improve learning and memory in Morris water maze test.	[58]
Catalpol	Aβ25-35 injected in rats intracerebroventricularly to establish AD model—5 or 10 mg/kg, i.p. for 7 days	More positive neurons (ChAT staining in cerebral cortex) and cells arranged in order; increase ChAT activity in dose dependent manner.	[28]
Catalpol	Orthotopic injection of Aβ25-35 into the right lateral ventricle of rats—5 and 10 mg/kg	Increase serum hydrocortisone level; decrease ACTH and CRH levels; alleviate structural damage of the hypothalamus.	[59]
Catalpol	Senescent mice treated with _D_-galactose—2.5, 5 or 10 mg/kg, subcutaneous for 2 weeks	Reverse the following senescence markers: increased AChE activity, decrease in ChAT positive neurons, decline in muscarinic AChR M1 (mAChR1) expression; increase in TNF-α, IL-1β) and AGEs levels.	[60]
Catalpol	Subcutaneously injected with _D_-galactose in mice—2.5, 5 or 10 mg/kg, subcutaneously for 2 weeks	Reverse cognition deficit and altered biochemical changes: increased LDH and decreased activities of GSH-ST, glutamine synthetase, creatine kinase in brain cortex and hippocampus.	[61,62]
Geniposide	APP/PS double transgenic AD mice model coupled with STZ-induced diabetes—5, 10, or 20 mg/kg intragastric for 4 weeks.	Decrease the concentrations of cerebral Aβ1-40 and Aβ1-42; up-regulate the protein levels of β-site APP cleaving enzyme (BACE1) and IDE; decrease the protein levels of ADAM10.	[63]
Geniposide	APP/PS1 doubly transgenic mice—12.5, 25 or 50 mg/kg, intragastric for 3 months	Ameliorate the Aβ1-42 induced decrease in synapse-related proteins (p-CaMKIIα/CaMKIIα, p-CREB/CREB, synaptophysin, and PSD-95) in neurons and APPswe/PS1dE9 mice; reverse the Aβ1–42 induced decrease in spine density on dendrites.	[64]
Geniposide	APP/PS1 AD transgenic mice—25 mg/kg for three months via intragastric administration	Improves learning and memory; suppresses the RAGE-dependent signaling (activation of ERK and IκB/NF-κB), production of TNF-α and IL-1β, and cerebral Aβ accumulation; augments synaptic plasticity by attenuating the Aβ-induced reduction of long-term potentiation and increasing the mEPSC amplitude and frequency in hippocampal neurons; reduces oxidative stress and mitochondrial dysfunction (increase the mitochondrial membrane potential).	[65,66]
Geniposide	STZ-induced AD model in rats—injection (50 μM, 10 μL) to the lateral ventricle	Prevent spatial learning deficit; reduce τ protein phosphorylation; elevate expression of GSK3β(pS-9) while suppressing GSK3β (pY-216); improve the altered neuronal ultrastructure.	[67]
Geniposide	Aβ1-42 in the hippocampus of STZ-induced diabetic rats. 12.5 or 25 mg/kg, intragastric for 46 days	Improve insulin and blood glucose; decrease Aβ1-42 level; improve the expression of IDE.	[68]
Linalool	Aβ1-40 (4 μg) solution injected in the bilateral hippocampus in mice—100 mg/kg, i.p.	Improve cognitive performance in Morris water maze test and step-through test; reverse the Aβ1-40 induced hippocampal cell injury in histological examination, apoptosis in TUNEL assay, changes of oxidative stress indicators (SOD, GPx, AChE); suppress the activated cleaved caspase (caspase-3, caspase-9) while elevating Nrf2, HO-1 expression.	[69]
Linalool	triple transgenic model of AD mice—25 mg/kg, p.o. every 48 h for 3 months	Improve learning and spatial memory and greater risk assessment behavior in the elevated plus maze; in the Hippocampi and amygdalae region, reduce extracellular β-amyloidosis, tauopathy, astrogliosis, microgliosis and pro-inflammatory markers (p38 MAPK, NOS-2, COX-2 and IL-1β).	[70]
Loganin	Scopolamine-induced AD model in rats—40 mg/kg, p.o.	Reverse shortening of step-through latency in the passive avoidance test; reduce the percent alternation in the Y-maze, and increased memory retention in the Morris water maze test.	[45]
Loganin	Scopolamine-induced AD model in mice—20 or 40 mg/kg, p.o. single dose	Reverse the memory impairment (Y-maze test; passive avoidance and the Morris water maze tests); inhibit AChE activity in the hippocampus and frontal cortex.	[71]
Oleuropein	Pentylenetetrazole-induced seizures in male NMRI mice–—10, 20 or 30 mg/kg; i.p.	Increased the seizure threshold; anticonvulsant effects reversed by naltrexone (opioid receptor antagonist).	[72]
Oleuropein	Colchicine (15 μg/rat) injected into the CA1 area of the hippocampus—10, 15 or 20 mg/kg, p.o. for 10 days	Improve learning and memory retention (Morris water maze test); attenuate the oxidative damage (assessed by GPx and CAT activities, nitric oxide and MDA).	[73]
Paeoniflorin	Transgenic mouse model of AD—2.0 mg/kg, i.p. for 24 h.	Improve cognitive function and ameliorate patterns of escape distance and escape latency in AD mice; decrease inflammation (protein expression levels of NF-κB, TNF-α, IL-1β, IL-6 and caspase-3 activity; inhibit cell death via increasing the Bcl-2/Bax ratio and p-Akt expression levels, and downregulating p-p38 MAPK expression in AD mice.	[74]
Paeoniflorin	Transgenic mouse model of AD—4 week treatment	Inhibit Aβ burden, Aβ-induced over activation of astrocytes and microglia; downregulate proinflammatory cytokines; upregulate anti-inflammatory cytokines in the brain; inhibit the activation of GSK-3β and reverse neuroinflammatory-induced NF-κB activation signaling pathways; exert inhibitory effects on NALP3 inflammasome, caspase-1, and IL-1β.	[75]
Paeoniflorin	Bilateral intrahippocampal injection of Aβ1-42 in rats—i.p. once daily for 14 days	Increased the expressions of Nrf2, HO-1 and γ-GCS mRNA; enhance the level of GSH and decrease the contents of MDA and carbonyl protein in the hippocampus; improve the NAIP expression and reduce the Caspase-3 expression in the hippocampus neurons.	[76]
Paeoniflorin	Aβ1-42-mediated neurotoxicity in rats—7.5, 15 or 30 mg/kg i.p. for 20 days	Improve memory (dose dependent) in Morris water maze test; inhibit neuronal apoptosis; maintain intracellular Ca^2+^ homeostasis; increase GSH content; suppress NOS activity and NO level, decrease of carbonyl protein and MDA levels.	[77]

ACTH, Adrenocorticotropic hormone; ADAM10, A disintegrin and metalloproteinase domain-containing protein 10; APP, amyloid precursor protein; APP/PS1 mice, double transgenic mice that over express the Swedish mutation of APP together with presenilin 1 deletion; CaMKII, calcium/calmodulin-dependent protein kinase II; CREB, cAMP-response element binding protein; CRH, corticotropin-releasing hormone; LDH, Lactate dehydrogenase; MDA, malondialdhyde; mEPSC, miniature excitatory postsynaptic current; NALP3, nacht domain-, leucine-rich repeat-, and pyrin domain (PYD)-containing protein 3; PSD-95, postsynaptic density protein 95.
